# The C-Terminus of Human Nucleotide Receptor P2X7 Is Critical for Receptor Oligomerization and N-Linked Glycosylation

**DOI:** 10.1371/journal.pone.0063789

**Published:** 2013-05-14

**Authors:** Lisa E. Wickert, Joshua B. Blanchette, Noelle V. Waldschmidt, Paul J. Bertics, John M. Denu, Loren C. Denlinger, Lisa Y. Lenertz

**Affiliations:** 1 Department of Biomolecular Chemistry, The University of Wisconsin-Madison, Madison, Wisconsin, United States of America; 2 Department of Medicine, The University of Wisconsin-Madison, Madison, Wisconsin, United States of America; 3 Department of Biology, St. Olaf College, Northfield, Minnesota, United States of America; New York University, United States of America

## Abstract

**Background:**

The P2X_7_ receptor binds extracellular ATP to mediate numerous inflammatory responses and is considered a potential biomarker and therapeutic target for diverse inflammatory and neurological diseases. P2X_7_ contains many single nucleotide polymorphisms, including several mutations located within its intracellular C-terminal trafficking domain. Mutations within the trafficking domain result in attenuated receptor activity and cell surface presentation, but the mechanisms by which amino acid changes within this region promote altered P2X_7_ function have not been elucidated.

**Methods and Results:**

We analyzed the amino acid sequence of P2X_7_ for any potential trafficking signals and found that P2X_7_ contains putative Arg-X-Arg ER retention sequences. Alanine substitutions near or within these sequences were constructed, and we determined that single mutation of R574 and R578 but not R576 or K579 attenuates P2X_7_-stimulated activation of ERK1/2 and induction of the transcription factors FosB and ΔFosB. We found that mutation of R578 within the trafficking domain to the naturally occurring Gln substitution disrupts P2X_7_ localization at the plasma membrane and results in R578Q displaying a higher apparent molecular weight in comparison to wild-type receptor. We used the glycosidase endoglycosidase H to determine that this difference in mass is due in part to the R578Q mutant possessing a larger mass of oligosaccharides, indicative of improper N-linked glycosylation addition and/or trimming. Chemical cross-linking experiments were also performed and suggest that the R578Q variant also does not form trimers as well as wild-type receptor, a function required for its full activity.

**Conclusions:**

These data demonstrate the distal C-terminus of P2X_7_ is important for oligomerization and post-translational modification of the receptor, providing a mechanism by which mutations in the trafficking domain disrupt P2X_7_ activity and localization at the plasma membrane.

## Introduction

The ATP receptor P2X_7_ is an important regulator of the inflammatory response and is considered a potential therapeutic target and biomarker for several inflammatory and neurological diseases [1]–[3]. P2X_7_ is a member of the P2X family of ionotropic nucleotide receptors (P2X_1−7_) and is expressed in several immune cells including monocytes, macrophages, and microglia [2], [4]. Numerous P2X_7_ knockout and pharmacological studies have demonstrated that P2X_7_ is important in glomerulonephritis, rheumatoid arthritis, cigarette smoke-induced lung inflammation, and depression. In addition, several groups are attempting to correlate P2X_7_ single nucleotide polymorphisms (SNPs) and/or P2X_7_ activity levels with viral-induced loss of asthma control, susceptibility to tuberculosis, and bipolar disorder [2]. Thus, P2X_7_ is gaining considerable interest as a pharmacological target for an array of diseases that have an inflammatory component.

P2X_7_ is activated by millimolar concentrations of ATP, which can be found in microenvironments with tissue damage or infected cells. ATP binding to this ion channel stimulates the influx and efflux of cations, activation of mitogen activated protein kinases (MAPKs), the processing of inflammatory mediators, gene transcription, and sometimes apoptosis [5], [6]. Specifically, activated P2X_7_ can stimulate the influx of Ca^2+^ and Na^+^ and the efflux of K^+^, the processing of interleukin-1β (IL-1β) into its mature form, and the generation of reactive oxygen species (ROS). P2X_7_ stimulation also results in activation of the MAPKs extracellular signal-regulated kinases 1/2 (ERK1/2) and the formation of a non-specific pore that allows the entry of molecules up to 900 Da [2], [6]. In addition, P2X_7_ agonists can activate and/or induce numerous transcription factors including cyclic-AMP response element-binding protein (CREB), nuclear factor-κB (NF-κB), and the activating protein-1 (AP-1) members JunB, FosB and ΔFosB. P2X_7_ ligands can also stimulate the expression of several immune mediators including IL-6, IL-8, vascular endothelial growth factor (VEGF), and cyclooxygenase-2 (COX-2) [2].

Although many studies have been performed to examine the contribution of P2X_7_ to normal physiology, relatively less research has been conducted to understand the basic mechanisms of P2X_7_ activity and how its domains regulate receptor function. P2X_7_ possesses a trafficking domain within its C-terminus, encompassing amino acids 551–590 [7], [8]. The trafficking domain contains several identified SNPs including R574H, R574C, R578G, R578Q, K579E, P582S, P582L, K583N and K583T (NCBI). We and others have demonstrated that mutations within the intracellular distal C-terminus of P2X_7_ result in altered trafficking, attenuated pore formation, and reduced cation channel activity [6], [7], [9]–[11]. Specifically, it has been reported that single mutations at I568, C572, R574, R578, F581, and double mutation of R578 and K579 promote decreased P2X_7_ cation channel and/or pore activity [7], [9], [10]. The aforementioned investigations have revealed that the wild-type residues are required for normal expression of P2X_7_ at the cell membrane, but the mechanisms by which these mutations result in attenuated activity have not been elucidated.

In this report we present evidence that mutation of the polymorphic residue R578 to Gln, a SNP identified in the SNP500Cancer project (NCBI), disrupts P2X_7_ oligomerization and results in improper addition and/or trimming of its N-linked glycosylation modifications, indicative of a potential processing defect in the endoplasmic reticulum (ER). According to the SNP500Cancer study, the R578Q polymorphism exhibits a heterozygosity frequency of 0.065 in Caucasians, but a larger data set is required to more accurately determine the frequency. In this study, we provide one of the first mechanisms to explain why the distal C-terminus of P2X_7_ is important for its plasma membrane localization and activity.

## Methods

### Reagents

The P2X_7_ agonist 2'(3')-*O*-(4-benzoylbenzoyl)-ATP (BzATP), tunicamycin, ionomycin, and phorbol 12-myristate 13-acetate (PMA) were obtained from Sigma (St. Louis, MO). Bis(sulfosuccinimidyl) suberate (BS^3^) and disuccinimidyl suberate (DSS) were obtained from Thermo Fisher Scientific (Waltham, MA), and endoglycosidase H (Endo H) was purchased from New England Biolabs (Ipswich, MA). Unless noted otherwise, the anti-P2X_7_ antibody was obtained from Santa Cruz Biotechnology (Santa Cruz, CA; cat. no. sc-25698), the anti-ERK1/2 antibody was purchased from Millipore (Billerica, MA; cat. no. 06–182), and the anti-vinculin antibody was from Sigma (cat. no. V4505). The anti-pERK1/2 antibody was purchased from Invitrogen (Carlsbad, CA; cat. no. 44680G), and the anti-FosB antibody was purchased from Cell Signaling Technology (Danvers, MA; cat. no. 2251). The anti-GRB2 antibody was purchased from Santa Cruz Biotechnology (cat.no. sc-255). The monoclonal anti-P2X_7_ antibody used in flow cytometry was from Dr. Iain Chessell (Glaxo Smith-Kline, Herts, U.K.) [10], and the anti-P2X_7_ antibody used in the EndoH assay was obtained from BD Biosciences (Franklin Lakes, NJ; discontinued). Alexa Fluor 488-labeled goat anti-mouse IgG was purchased from Life Technologies (Grand Island, NY; cat. no. A-11029), and PE-labeled mouse IgG2b isotype control was purchased from BD Biosciences (cat. no. 555058).

### Cell Culture and Harvesting

HEK293 (American Type Culture Collection, Manassas, VA) were cultured in Dulbecco’s modified Eagle’s medium (DMEM) supplemented with 10% cosmic calf serum (CCS) (Mediatech, Herndon, VA), 1% L-glutamine, and 100 U/ml penicillin/streptomycin. Human monocytes were purified and cultured as described before [12]. The cells were incubated at 37°C under 5% CO_2_. Cell lysates were generally prepared by harvesting the cells in 2X sample buffer (20 mM Tris-HCl pH 6.8, 2 mM EDTA, 2 mM DTT, 1 mM Na_3_VO_4_, 2% SDS, 20% glycerol, 0.2% bromophenol blue) followed by boiling and sonicating the samples.

### P2X_7_ Constructs

Full-length human P2X_7_ (accession number AAH11913) was subcloned into pcDNA3. The P2X_7_ point mutants were generated by site-directed mutagenesis using the following primers and their reverse complements. For the double mutation, the N-terminal amino acid was mutated first followed by mutation of the second residue.

P2X7 R469A 5′-GAAAGGAGGCGACTCCTGCATCCAGGGATAGC - 3′.

P2X7 R469A/R471A 5′- CTTAGAAAGGAGGCGACTCCTGCATCCGCGGATAGCC - 3′.

P2X7 R544A 5′- GATTCCACCAACAGCGCGCTGCGGCACTGTG - 3′.

P2X7 R574A 5′- CCAGCTGCTGCGCCTGGAGGATCC - 3′.

P2X7 R576A 5′- CTGCTGCCGCTGGGCGATCCGGAAAG - 3′.

P2X7 R578A 5′- CTGCCGCTGGAGGATCGCGAAAGAGTTTC - 3′.

P2X7 R578Q 5′- CTGGAGGATCCAGAAAGAGTTTCCGAA - 3′.

P2X7 K579A 5′- GGAGGATCCGGGCAGAGTTTCC - 3′.

### Generation of Stable Cell Lines

HEK293 cells were transfected with pcDNA3 containing wild-type or mutant P2X_7_ using FuGENE™ 6 transfection reagent according to the manufacturer’s instructions (Roche). The cells were then selected with 500 µg/mL G418 sulfate (Sigma) to obtain a resistant population.

### YO-PRO-1 Dye Uptake Assay

HEK293 cells transiently or stably expressing the indicated constructs were cultured on glass cover slips coated with 0.01% poly-*l*-lysine (Sigma). The transient-expressing cells were transfected using FuGENE™ 6 transfection reagent according to the manufacturer’s instructions. The cells were stimulated at room temperature in potassium-glutamate buffer (130 mM K-glutamate, 20 mM HEPES-KOH (pH 7.4), 5 mM KCl, 0.1% BSA, 10 mM glucose) with 250 µM BzATP or HEPES buffer control for 10 min in the presence of 1 µM YO-PRO-1 (Molecular Probes, Eugene, OR). Potassium-glutamate buffer is known to facilitate robust YO-PRO-1 uptake in response to BzATP, as previously reported [13], [14]. The cells were then treated with 10 mM MgCl_2_ to close the pore, fixed with 4% paraformaldehyde and imaged with a Zeiss Axioplan 2 microscope at 63X magnification.

### ERK1/2 Activation and FosB/ΔFosB Induction Assays

HEK293 cells were cultured in collagen I-coated wells. Cells for the ERK1/2 activation tests were stimulated with 250 µM BzATP or buffer control for 5 min and lysates were prepared as performed previously [15]. Similar to our previous study, cells for the FosB/ΔFosB induction assays were treated with 250 µM BzATP or buffer control for 5 min, the medium was replaced, and the cells were harvested 2 h later [12]. Proteins from cell lysates were separated by electrophoresis on 10% sodium dodecyl sulfate (SDS)-polyacrylamide gels, transferred to polyvinylidene fluoride (PVDF) membranes and blocked in 5% nonfat dry milk. The membranes were probed with the indicated primary antibodies, incubated with secondary antibodies conjugated to horseradish peroxidase (Santa Cruz Biotechnology), and visualized by enhanced chemiluminescence. The fold changes in ERK1/2 activation and FosB/ΔFosB induction were determined using Adobe Photoshop CS4 Version 11.0 to compare the relative band intensities of the BzATP-stimulated samples with the buffer control-stimulated samples. The background intensity was subtracted from the value of the pERK1/2 and FosB/ΔFosB bands, and these adjusted values were normalized to a loading control prior to calculating the fold changes. For quantifying the relative levels of pERK1/2, the band intensities of both pERK1 and pERK2 were used in the calculations. For the FosB/ΔFosB calculations, the band intensities of full-length FosB and the observed ΔFosB species were included. ΔFosB reportedly exists as a phosphorylated protein [16].

### Proteinase K Digestion Assay

HEK293 stable cell lines were cultured in 10 cm plates to subconfluency. The cells were then lifted from the plates by rinsing them with serum-free medium and pelleted at 200×*g.* The cells were gently suspended in serum-free DMEM and incubated with 200 µg/mL proteinase K at 37°C for 90 min. The samples were boiled for 10 min to inactivate proteinase K, 2X sample buffer was added, and the lysates were sonicated. The generation of the ∼240 amino acid P2X_7_ intracellular C-terminus was assessed by immunoblotting the lysates with an anti-P2X_7_ antibody that was raised against the C-terminus of P2X_7_.

### Chemical Cross-linking

P2X_7_ monomers were cross-linked using protocols modified from Boumechache *et al.* [17]. To cross-link protein from cell lysates, HEK293 stable cell lines were cultured to subconfluency and harvested in phosphate buffer saline (PBS) containing 1% Triton X-100. The cells were incubated on ice for 30 min, sonicated and centrifuged at 21,000 x *g* to remove the Triton-insoluble material. The samples were incubated with 500 µM BS^3^ at room temperature for 30 min, and the reaction was quenched by the addition of 20 mM Tris HCl pH 7.5. An equal volume of 2X sample buffer was added, and ∼50 µg of protein from the lysates were boiled for 2 min. The proteins were separated on 7.5% polyacrylamide gels containing SDS, transferred to PVDF membrane, and immunoblotted with anti-P2X_7_ antibody to detect P2X_7_ trimers.

To cross-link proteins expressed at the plasma membrane, HEK293 stable cell lines were cultured to subconfluency and treated with 500 µM BS^3^ at room temperature or 37°C for 30 min. The cells were harvested in 2X sample buffer, sonicated, boiled, and immunoblotted as described above.

To cross-link intracellular proteins, HEK293 stable cell lines were treated with 500 µM DSS at 37°C for 30 min. The reaction was quenched by the addition of 20 mM Tris HCl pH 7.5, and the proteins were processed as described above.

The differences in the amount of wild-type P2X_7_ and P2X_7_ R578Q that formed trimers upon cross-linking were calculated and compared. Adobe Photoshop CS4 Version 11.0 was used to obtain the relative band intensities of the P2X_7_ monomers, P2X_7_ trimers, and vinculin (loading control). The P2X_7_ trimer values were normalized to the relative expression levels of the monomers. The adjusted values for the P2X_7_ trimers were then normalized to vinculin, and Student’s *t*-tests were performed to determine the statistical significance between the normalized levels of the P2X7_7_ wild-type and P2X_7_ R578Q trimers.

### Flow Cytometry

HEK293 stable cell lines were cultured in 10 cm plates to subconfluency. The cells were lifted by brief exposure with 0.25% trypsin, pelleted at 200×*g*, and suspended in 1X PBS pH 7.4 containing 1% CCS. 5×10^5^ cells were treated with 1 µg/µl anti-P2X_7_ or 1 µg/µl IgG2b isotype control for 30 min at 4°C. The cells were washed with 1 mL of 1% CCS PBS and suspended in 100 µl 1% CCS PBS. Secondary labeling of cells was performed with 1 µg/µl Alexa Fluor 488-conjugated goat anti-mouse IgG for 30 min at 4°C. After an additional wash, the cells were suspended in 200 µl 1% CCS PBS. To exclude dead cells, 3 µg/ml propidium iodide was added to each sample. A total of 10,000 events were collected on a FACSCalibur flow cytometer (BD Biosciences) at the University of Wisconsin Carbone Cancer Center Flow Cytometry Core Facility. The data were analyzed with FlowJo analysis software (TreeStar, OR).

### Cell Viability Assay

HEK293 stable cell lines that were treated with BS^3^, DSS or tunicamycin were incubated with 0.2% trypan blue and visualized by microscopy. All of the cells in one field of view (∼70 cells) were counted, the number of trypan blue-positive cells was determined, and the percent viability was calculated.

### Endo H glycosidase Assay

HEK293 stable cell lines were harvested in 2X sample buffer, and the proteins were denatured by boiling for 10 min. The cell lysates were typically treated with 5 U EndoH in 50 mM sodium citrate (pH 5.5) at 37°C for 1 h. The proteins (∼50 µg) were then resolved on 12.5% polyacrylamide gels containing SDS, transferred to PVDF membranes, and immunoblotted with an anti-P2X_7_ antibody.

### Tunicamycin Assay

HEK293 stable cell lines were pretreated with 1 µg/ml of tunicamycin for approximately 48 h and then incubated with 500 µM DSS for 15 min prior to quenching by the addition of 20 mM Tris HCl pH 7.5. The cells were harvested in 2X sample buffer, sonicated, boiled and immunoblotted as described above. To compare the relative levels of trimers that formed with and without tunicamycin treatment, Adobe Photoshop CS4 Version 11.0 was used to obtain the relative band intensities of the P2X_7_ monomers, P2X_7_ trimers, and vinculin (loading control). The P2X_7_ trimer values were normalized to the relative expression levels of the monomers, and the adjusted values for the P2X_7_ trimers were then normalized to vinculin.

## Results

### P2X_7_ amino Acids Arg 574 and Arg 578 within the Trafficking Domain are Critical for Receptor Activity

**Figure 1:**
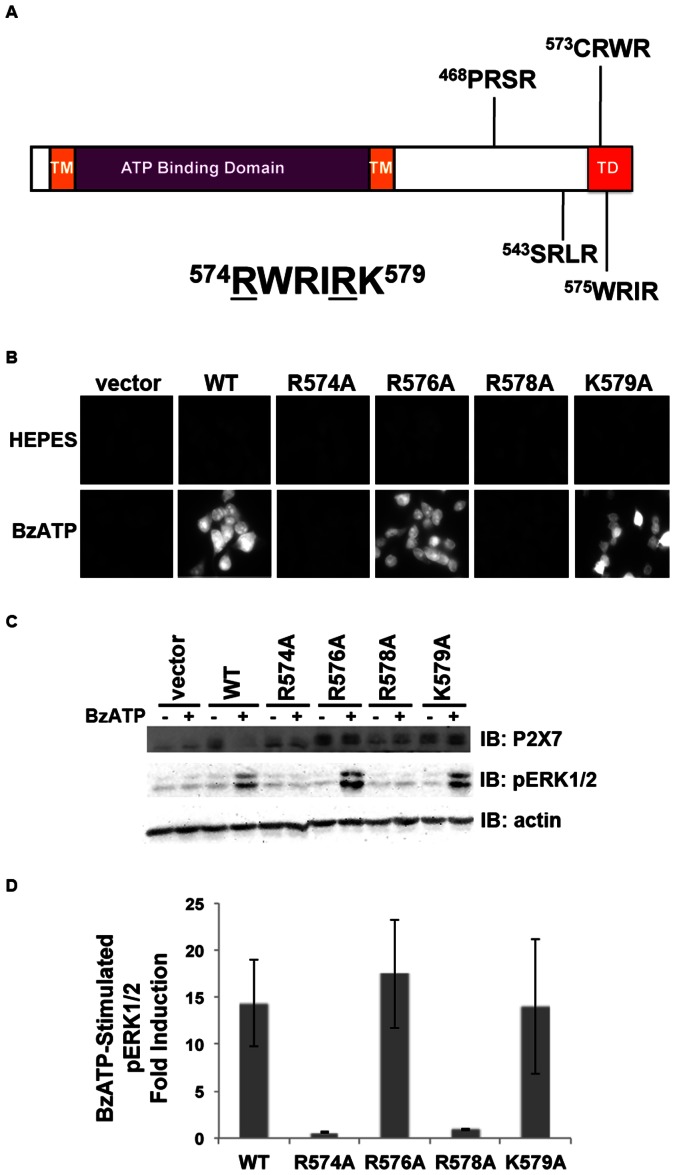
Amino acids Arg574 and Arg578 within the trafficking domain (and potential ER retention sequences) are critical for P2X_7_ activity. **A,** Human P2X_7_ contains two transmembrane domains, an extracellular ATP binding domain, and an intracellular C-terminal domain containing a region important for cell surface localization (TD). The receptor also contains four potential Arg-X-Arg ER retention sequences beginning at amino acids 468, 543, 573 and 575 [18]. The underlined amino acids were found to be important for P2X_7_ activity. TM = transmembrane domain, TD = trafficking domain. **B,** HEK293 cells expressing P2X_7_ R574A or R578A but not the R576A or K579A mutants exhibit attenuated BzATP-stimulated pore formation. Cells were treated for 10 min with 250 µM BzATP in the presence of the fluorescent dye YO-PRO-1. The pores were closed by the addition of MgCl_2_, and the cells were imaged for relative YO-PRO-1 uptake. These data are representative of at least four experiments. **C and D,** The P2X_7_ R574 and R578 mutants but not the R576A and K579A mutants have an attenuated ability to activate ERK1/2 upon nucleotide stimulation. The lower band observed in every lane on the P2X_7_ immunoblot is a non-specific protein; the top band is exogenous P2X_7_. The error bars represent the standard error of the mean. These data are representative of at least three experiments.

We previously demonstrated that the distal C-terminus of human P2X_7_ is critical for its activity and localization at the plasma membrane [10]. Specifically, we generated the P2X_7_ R578E/K579E double mutant to investigate its conserved lipopolysaccharide (LPS) binding domain and found that mutation of R578 and K579 results in attenuated P2X_7_-stimulated ion currents, pore formation and apoptosis [8], [10]. We analyzed the sequence of the intracellular C-terminus to identify motifs that may regulate trafficking and found that P2X_7_ contains four RXR sequences, motifs for the poorly understood Arg-based ER retention signals (Fig. 1a). Arg-based ER retention signals reportedly conform to the sequence Φ/Ψ/R-R-X-R where Φ/Ψ represents an aromatic or bulky hydrophobic residue and X is any amino acid [18]. We previously postulated that human P2X_7_ contains an Arg-based ER retention sequence at amino acids 576–578 and demonstrated that double mutation of R576 and R578 to Ala results in attenuated P2X_7_ ligand-stimulated pore formation [6]. Here we sought to determine which specific residues in the Arg-X-Arg sequences within the trafficking domain modulate P2X_7_ function. We tested the ability of alanine substitutions to stimulate P2X_7_ ligand-induced pore formation and ERK1/2 activation. As shown in Figure 1, mutation of R574 or R578 but not mutation of R576 or K579 results in attenuated BzATP-stimulated pore formation and ERK1/2 phosphorylation/activation. These results indicate that R574 and R578 but not R576 and K579 are important for P2X_7_ activity.

### The P2X_7_ Arg578 to Gln Polymorphism Exhibits Attenuated BzATP-induced Signaling and Pore Formation

**Figure 2:**
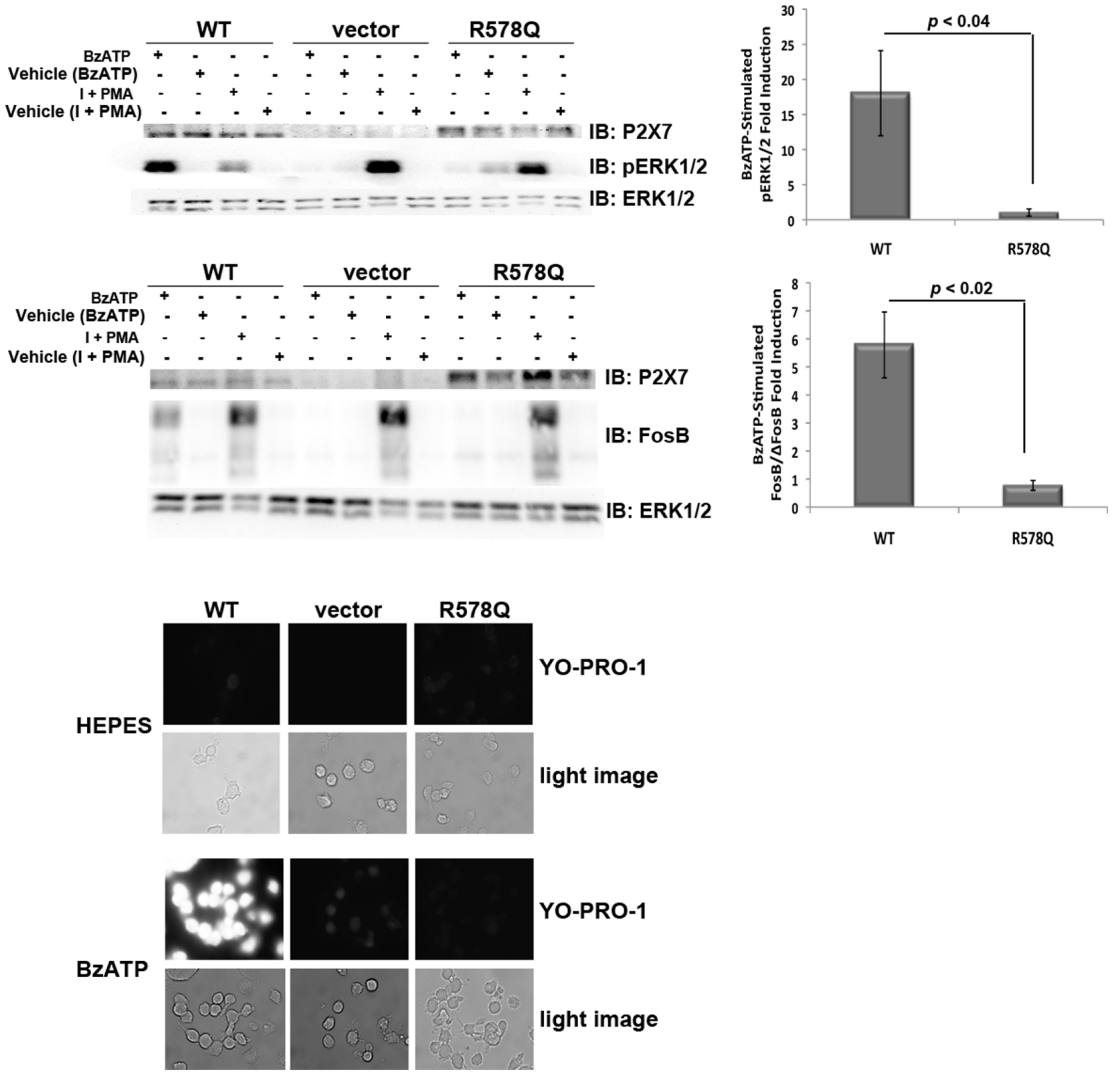
The P2X_7_ R578Q variant exhibits attenuated nucleotide-stimulated signaling and pore formation. **A and B,** HEK293 cells expressing the P2X_7_ R578Q variant exhibit attenuated ERK1/2 activation. We have previously found that treatment with 1 µg/mL PMA plus 1 µg/mL ionomycin promotes ERK1/2 activation (data not shown), and this co-treatment was used here as a positive control. The error bars represent the standard error of the mean. These data are representative of at least three experiments. **C and D,** HEK293 cells expressing the P2X_7_ R578Q variant display decreased nucleotide-induced FosB/ΔFosB induction in comparison to wild-type P2X_7_. We have previously found that treatment with 1 µg/mL PMA plus 1 µg/mL ionomycin stimulates FosB/ΔFosB induction (data not shown), and this co-treatment was used here as a positive control. The top band on the FosB immunoblot is full-length FosB and the lower bands are its truncated splice variant ΔFosB [27]. The error bars represent the standard error of the mean. These data are representative of at least three experiments. **E,** The P2X_7_ R578Q variant displays drastically attenuated pore formation. These images are representative of at least three experiments. I = ionomycin.

To determine whether a naturally-occurring variant within the P2X_7_ trafficking domain exhibits attenuated activity, we examined BzATP-stimulated ERK1/2 phosphorylation/activation, FosB/ΔFosB induction, and pore formation in HEK293 cells stably-expressing P2X_7_ R578Q [6], [12], [15]. The P2X_7_ R578Q mutation is more structurally similar to Arg compared to Ala. As shown in Figure 2, mutation of R578 to Gln results in attenuation of both signaling (ERK1/2 phosphorylation/activation and FosB/ΔFosB induction) and pore activity. The R578Q variant resulted in approximately a 17-fold reduction in ERK1/2 activation and approximately a 6-fold reduction in FosB/ΔFosB induction in comparison to P2X_7_ wild-type. The cells were treated with PMA and ionomycin as a positive control to verify that introduction of P2X_7_ R578Q does not result in a global inability to activate ERK1/2 or induce FosB/ΔFosB. It is worth noting that throughout our studies the relative expression levels between P2X_7_ wild-type and the R578Q mutant were typically within a 1.5 to 2-fold difference. These results show that a P2X_7_ distal C-terminal variant found in the human population also displays reduced receptor function.

### P2X_7_ R578Q does not Stably Express on the Cell Surface

**Figure 3:**
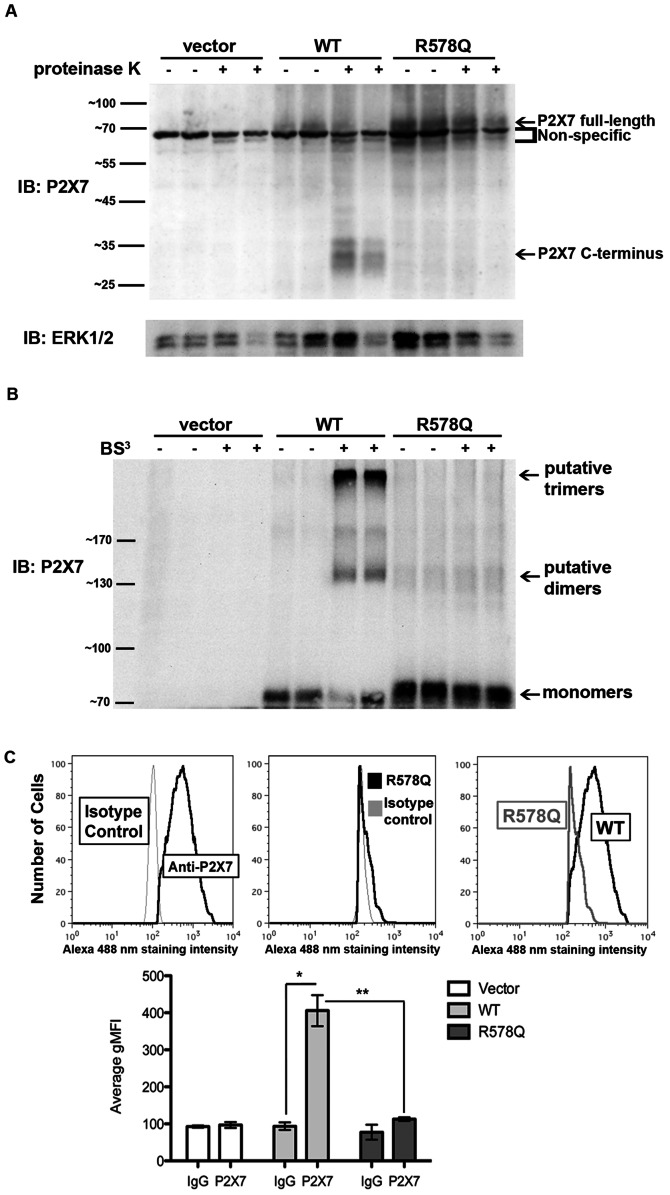
P2X_7_ R578Q is not expressed on the plasma membrane. **A,** HEK293 stable cell lines were treated with the broad-spectrum protease proteinase K to detect the presence of the ∼30 kDa P2X_7_ C-terminal tail that is liberated when the extracellular domain of P2X_7_ is digested by protease when the receptor is localized at the plasma membrane. These data are representative of three experiments. **B,** HEK293 stable cell lines were treated with the cell-impermeable chemical cross-linker BS^3^ to detect P2X_7_ localization at the cell surface. The P2X_7_ monomers and putative dimers and trimers are indicated. The predicted molecular weight of human P2X_7_ is ∼69 kDa and it contains several N-linked glycosylation sites that result in slower mobility in SDS-PAGE gels [15]. Thus, P2X_7_ dimers are predicted to exhibit an apparent mass slightly greater than 140 kDa and P2X_7_ trimers are predicted to exhibit an apparent mass slightly greater than 210 kDa. These data are representative of at least four experiments. **C,** HEK293 cells were stained with an anti-P2X_7_ antibody or IgG isotype control, and the relative levels of surface-exposed P2X_7_ WT and R578Q were determined by flow cytometry. The graph represents the average of three independent experiments. * *p*<0.006, ** *p*<0.007. gMFI = geometric mean fluorescence intensity.

To assess the mechanism by which P2X_7_ R578Q exhibits reduced function, we used a protease digestion assay that we previously developed [15] to demonstrate that there is no detectable P2X_7_ R578Q expressed on the plasma membrane (Figure 3a). In this assay, live cells are exposed to the broad-spectrum protease proteinase K for a given period of time. The extracellular domain of P2X_7_ is digested by protease when the receptor is localized at the plasma membrane, liberating the intracellular C-terminal tail that can be detected by immunoblotting using antibodies raised against the C-terminus. The data in Figure 3a demonstrate that the P2X_7_ R578Q polymorphism exhibits a drastically reduced ability to properly localize.

**Figure 4:**
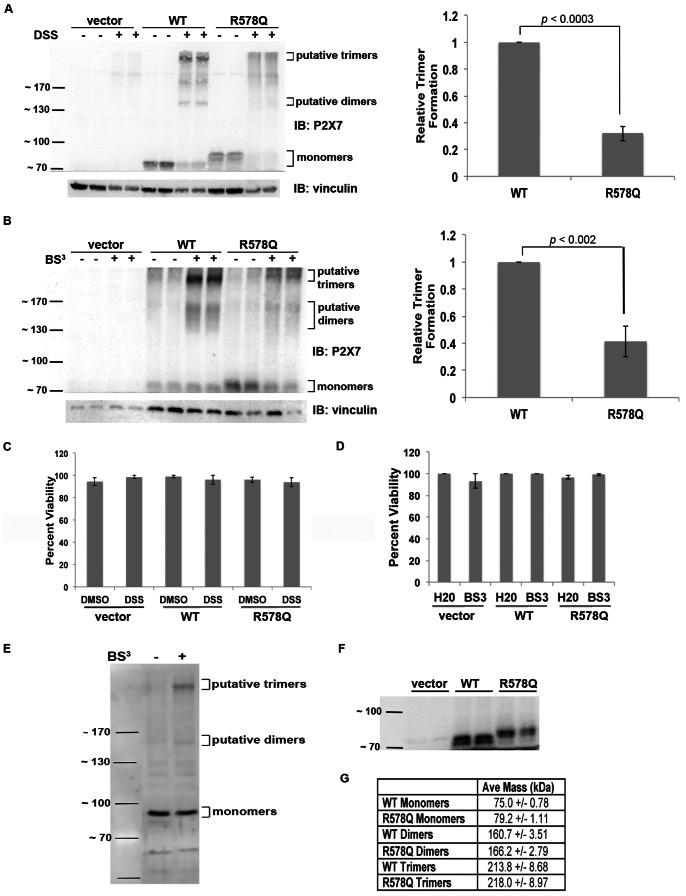
P2X_7_ R578Q displays defective oligomerization. **A,** HEK293 cells were treated with the cell-permeable chemical cross-linker DSS to demonstrate that intracellular P2X_7_ R578Q does not oligomerize as efficiently as wild-type receptor. The samples were blotted with anti-vinculin antibody as a loading control. The average difference in the amount of P2X_7_ wild-type trimers that formed following treatment with DSS was calculated and compared to the amount of P2X_7_ R578Q trimers that formed. A Student’s *t*-test was performed to determine statistical significance. These data are representative of five experiments. **B,** Proteins from HEK293 lysates were cross-linked with BS^3^, and the difference in the amount of P2X_7_ wild-type trimers that formed upon cross-linking with BS^3^ was compared to the amount of P2X_7_ R578Q trimers that formed. A Student’s *t*-test was performed to determine statistical significance. These data are representative of five experiments. **C,D,** HEK293 cells were incubated in the presence of DSS or BS^3^, respectively, or the appropriate vehicle control. The cells were stained with trypan blue and counted to determine the percent viability. The error bars represent the standard error of the mean. These data are representative of three experiments. **E,** Human monocytes were treated with BS^3^ to demonstrate that upon cross-linking, endogenous P2X_7_ displays a banding pattern that is consistent with dimer and trimer formation. These data are representative of four experiments. **F,** Proteins from untreated lysates were resolved on 12.5% SDS-polyacrylamide gels to demonstrate the apparent size difference between P2X_7_ wild-type and R578Q. These data are representative of at least fifteen experiments. **G,** The molecular masses of the P2X_7_ wild-type and R578Q monomers and their putative dimers and trimers were extrapolated using the distance the molecular weight standards migrated. The results were averaged and the standard error of the mean was determined. Seven replicates were used to calculate the average mass of the monomers, and three replicates were used to calculate the mass of the dimers and trimers.

To further verify that P2X_7_ R578Q does not stably express at the plasma membrane, we used a chemical cross-linker to examine the presence of P2X_7_ trimers at the cell surface. Intact cells were treated with BS^3^, a hydrophilic, cell impermeable, non-cleavable cross-linker [19], and the presence of P2X_7_ trimers was assess by immunoblotting. Many groups have reported that P2X_7_ exists as a homotrimer [4], [17], [20]–[22]. As shown in Figure 3b, cross-linking proteins on live cells promotes the detection of putative P2X_7_ wild-type trimers but not the detection of P2X_7_ R578Q oligomers. The mass of the multimeric complex for wild-type P2X_7_ is consistent with P2X_7_ homotrimer formation (Figure 4g).

We also utilized flow cytometry to demonstrate that P2X_7_ wild-type but not P2X_7_ R578Q is expressed on the plasma membrane of HEK293 cells (Fig. 3c). There was a statistically significant difference in the amount of wild-type receptor observed at the cell surface in comparison to the amount of the R578Q variation present (*p*<0.007). In addition to demonstrating that P2X_7_ R578Q trimers are not stably expressed at the cell surface, these results demonstrate that wild-type P2X_7_ constitutively exists as a trimer at the plasma membrane and does not require ligand stimulation to oligomerize.

### The P2X_7_ R578Q SNP Displays Defective Oligomerization

To further delineate the mechanism by which P2X_7_ R578Q possesses reduced function, we tested the idea that this mutant displays defective oligomerization. We cross-linked proteins from both live cells and cell lysates and demonstrate that R578Q does not oligomerize as efficiently as wild-type receptor (Fig. 4). In Figure 4a, we treated live cells with the cell permeable cross-linker DSS and observed that treatment with DSS results in the formation of less P2X_7_ R578Q trimers in comparison to wild-type (*p*<0.0003). To validate these results, cell lysates were treated with BS^3^ (Fig. 4b). As with the live cells, we observed that the R578Q variant forms less trimers upon cross-linking in comparison to wild-type receptor (*p*<0.002). Thus, we have presented evidence that the P2X_7_ R578Q mutant does not exist as a trimeric complex at the cell surface (Fig. 3b), a significantly larger portion of intracellular P2X_7_ wild-type receptor exists as trimers in comparison to intracellular P2X_7_ R578Q trimers (Fig. 4a), and the total amount of wild-type receptor that oligomerizes is greater than the total amount of P2X_7_ R578Q that oligomerizes (Fig. 4b).

To examine the possibility that treatment with the chemical cross-linkers induces cell death and potential protein-protein interactions that do not occur in normal, intact healthy cells, we performed cell viability assays. As shown in Figures 4c and 4d, there was no observable cell death that occurs in the presence of DSS or BS^3^, respectively.

To further support our interpretation that the ∼210 kDa bands observed upon cross-linking HEK293 cells are P2X_7_ trimers, we cross-linked proteins from human monocytes and observed that endogenous P2X_7_ also appears to trimerize upon treatment with BS^3^ (Fig. 4e).

In addition, we observed that when P2X_7_ wild-type and the R578Q mutant are resolved on 12.5% polyacrylamide gels, it becomes more apparent that the R578Q monomers migrate slower than wild-type protein (Fig. 4f). This suggests that P2X_7_ R578Q may contain a larger mass of post-translational modifications than wild-type P2X_7_. We used the molecular weight standards to extrapolate the masses of the monomers, dimers, and trimers of P2X_7_ wild-type and P2X_7_ R578Q (Fig. 4g). As predicted, the monomers of both P2X_7_ wild-type and R578Q exhibited an apparent molecular weight greater than the calculated weight of 69 kDa (no post-translational modifications), the dimers have an apparent weight greater than 140 kDa, and the trimers have an apparent mass that is greater than 210 kDa. Interestingly, the monomers, dimers, and trimers of the P2X_7_ R578Q mutant have a higher calculated mass than the monomers, dimers, and trimers of wild-type receptor.

### P2X_7_ R578Q is More Heavily Glycosylated than Wild-type P2X_7_

**Figure 5:**
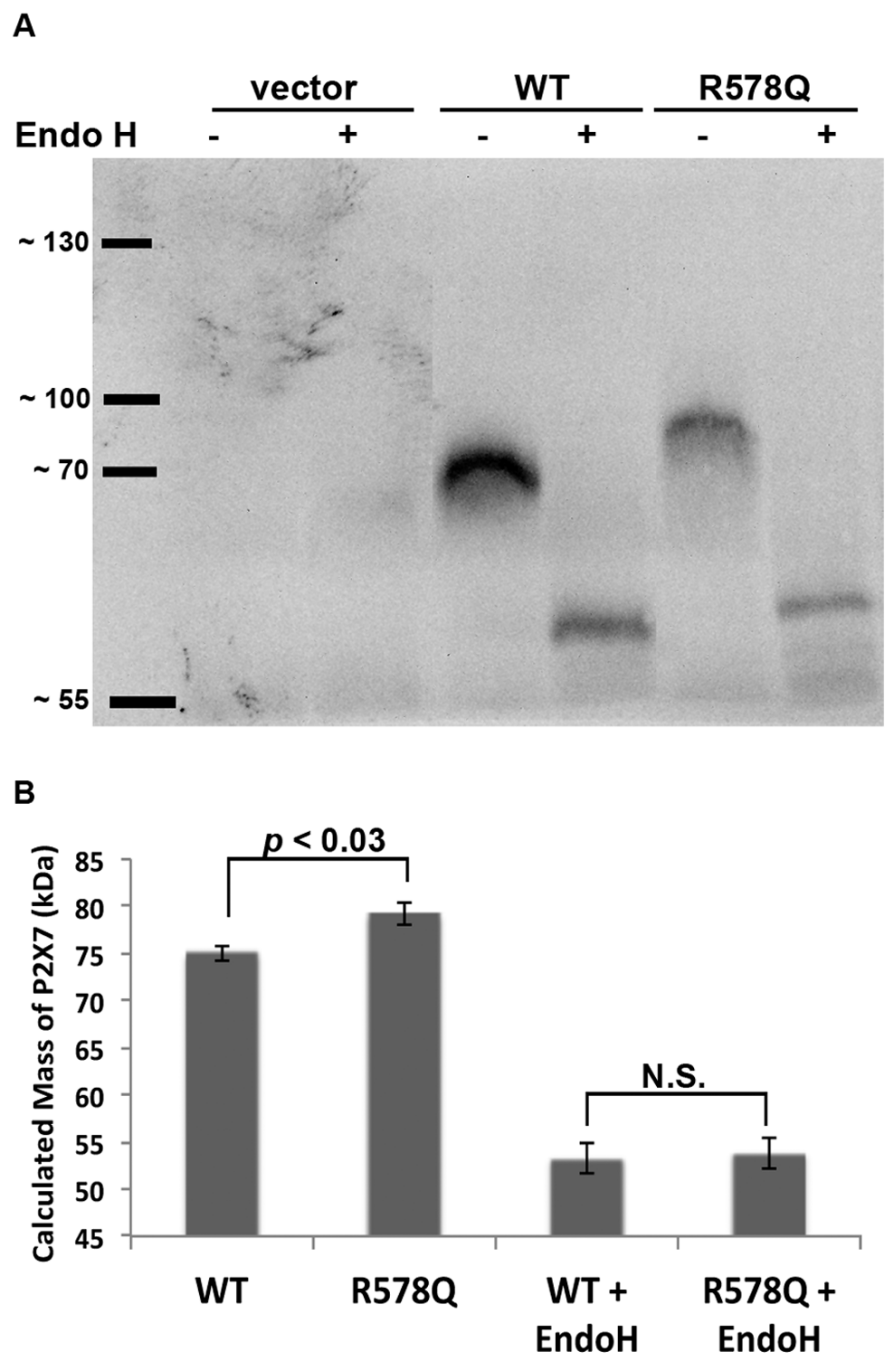
P2X_7_ R578Q contains a larger mass of N-linked glycosylation modifications than wild-type receptor. **A,** Proteins from cell lysates were treated with the glycosidase Endo H and compared with the mobility of proteins from untreated lysates. The size difference between glycosylated P2X_7_ wild-type and P2X_7_ R578Q appears to be larger than the size difference between the deglycosylated receptors. These data are representative of three experiments. **B,** The molecular mass of untreated and Endo H-treated P2X_7_ wild-type and P2X_7_ R578Q were calculated using the distance the molecular weight standards migrated. The error bars represent the standard error of the mean. Seven replicates were used to calculate the mass of untreated P2X_7_, and three replicates were used to calculate the mass of Endo H-treated P2X_7_. N.S. = not statistically significant.

To test the hypothesis that the R578Q mutant possesses a larger mass of N-linked glycosylation modifications, we compared the relative molecular masses of P2X_7_ wild-type and R578Q both before and after deglycosylation by Endo H. We previously reported that P2X_7_ is N-linked glycosylated on N187, N202, N213, N241 and N284 [15]. As shown in Figures 4f and 5, wild-type P2X_7_ migrates faster than the R578Q mutant. Based upon SDS-PAGE gel analysis, treatment with Endo H results in a decrease in mass, and the molecular masses of P2X_7_ wild-type and the R578Q variant are approximately the same following deglycosylation (Fig. 5a). The molecular mass of P2X_7_ wild-type and the R578Q variant both before and after deglycosylation were calculated and compared (Fig. 5b). There was a statistically significant difference in the sizes of the untreated receptors, but there was no apparent difference in the size of the receptors that were deglycosylated with Endo H. The mass of P2X_7_ wild-type was calculated to be 75.0 kDa and the mass of P2X_7_ R578Q was calculated to be 79.2 kDa. The difference in mass between deglycosylated wild-type and deglycosylated mutant receptor is drastically smaller; deglycosylated wild-type P2X_7_ has a calculated mass of 53.3 kDa while P2X_7_ R578Q has a calculated mass of 53.8 kDa. Of note, human P2X_7_ without any post-translational modifications has a predicted molecular mass of approximately 69 kDa, but upon deglycosylation the protein may be folded in a manner that allows faster migration through SDS-PAGE gels, causing the receptor to migrate at approximately the same position as the 55 kDa standard [15].

### N-linked Glycosylation and Oligomerization of P2X_7_ are Independent Events

**Figure 6:**
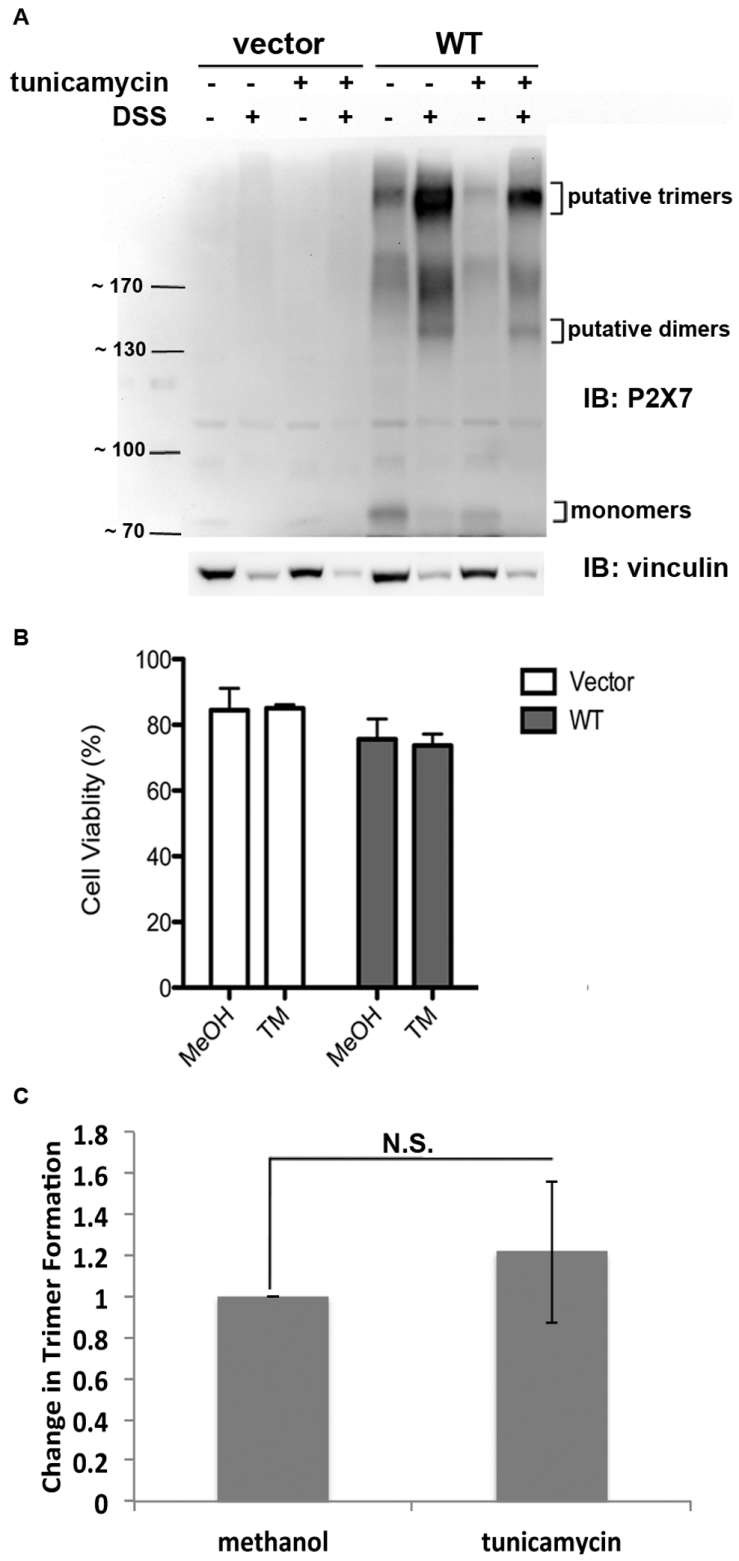
Oligomerization of P2X_7_ is independent of glycosylation. **A,** Protein was cross-linked following treatment with the N-linked glycosylation inhibitor tunicamycin and immunoblotted to detect the formation of P2X_7_ wild-type trimers. **B,** A cell viability assay was performed to determined whether tunicamycin induces cell death. These data are the average of three independent experiments, and the error bars represent the standard error of the mean. MeOH = methanol, TM = tunicamycin. **C,** The results were quantified as described above and indicate that there was no observable difference in the amount of trimers that formed with or without tunicamycin. These data represent the average of five independent experiments. N.S. = not statistically significant.

To determine the significance of N-linked glycosylation in P2X_7_ oligomerization, we inhibited N-linked glycosylation via treatment with tunicamycin and assessed the ability of P2X_7_ wild-type to cross-link (Fig. 6). Because fewer P2X_7_ monomers and P2X_7_ trimers were observed following tunicamycin treatment (Fig. 6a), we performed cell viability assays to assess whether tunicamycin induces cell death (Fig. 6b). Cells treated with 1 µg/ml tunicamycin or methanol vehicle control for 48 h exhibited little difference in cell viability. Thus, the differences in trimer formation with or without tunicamycin were normalized to the relative levels of monomers that were observed following treatment with methanol or tunicamycin for 48 h (Fig. 6c). Student’s *t*-tests were performed to determine whether there were statistically significant differences, and no differences were observed (*p* = 0.57). Thus, after normalizing the levels of P2X_7_ trimers to the amount of monomers present, treatment with tunicamycin for 48 h does not appear to attenuate the ability of P2X_7_ to oligomerize.

### Alanine Mutations within the Arg-X-Arg Sequences Located at Amino Acids 469 and 544 do not Result in Altered P2X_7_ Activity

**Figure 7:**
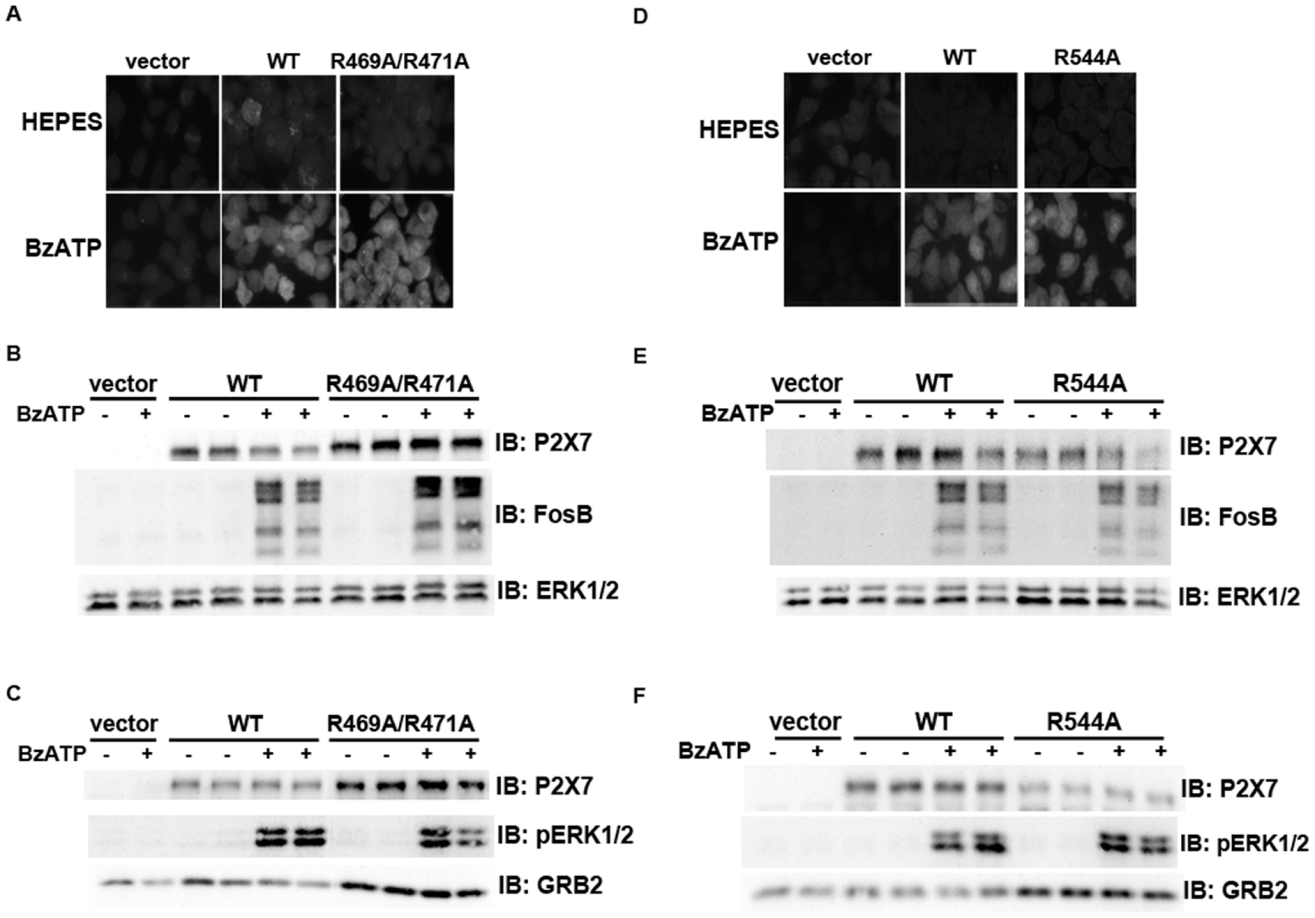
Mutations within the other Arg-X-Arg sequences do not result in altered P2X_7_ activity. **A and D,** The P2X_7_ R469A/R471A double mutant and the P2X_7_ R554A single mutant do not display attenuated nucleotide-stimulated pore activity. These data are representative of at least three experiments. **B and E,** The P2X_7_ R469A/R471A and R554A mutants do not display attenuated BzATP-stimulated FosB/ΔFosB induction. The anti-FosB antibody recognizes both full-length FosB (indicated by the top three bands) and its truncated splice variant ΔFosB (indicated by the bottom two bands). These data are representative of at least two experiments. **C and F,** The P2X_7_ R469A/R471A and R554A mutants exhibit normal nucleotide-stimulated ERK1/2 activation. These data are representative of two experiments.

To determine whether the RXR sequences located at amino acids R469 and R544 are also important for receptor activity (Fig. 1a), we generated alanine substitutions at these motifs and assessed BzATP-stimulated pore formation and cell signaling. As shown in Figure 7, P2X_7_ R469A/R471A and P2X_7_ R544A both exhibit BzATP-stimulated pore formation, FosB/ΔFosB induction and ERK1/2 phosphorylation/activation that is similar to that observed for wild-type P2X_7_. The fold changes in FosB/ΔFosB induction and ERK1/2 activation by P2X_7_ wild-type, R469A/R471A and R544A were calculated and compared. The differences in FosB/ΔFosB induction between P2X_7_ wild-type and P2X_7_ R469A/R571A differed within 1.5 fold, and the differences between P2X_7_ wild-type and R544A differed within 1.3 fold. As for pERK1/2 activation, the differences between wild-type and R469A/R471A differed within 1.7 fold and 1.5 for wild-type and R544A.

## Discussion

We present evidence that residue R578 in human P2X_7_ is important for processing newly synthesized P2X_7_ in the ER where N-linked glycosylation addition/trimming and oligomerization occurs [23]. Although we and other groups have demonstrated that mutations in the distal C-terminus of P2X_7_ result in attenuated activity and cell surface presentation [6], [7], [9], [11], this is the first report to our knowledge that describes a possible mechanism by which amino acid changes in this region result in reduced function. Our data indicate there is an apparent size difference between wild-type P2X_7_ and the R578Q variant, and this mass difference is attributed in part to the presence of increased oligosaccharides on the R578Q mutant. This result supports the idea that mutation of R578 prevents the proper addition and/or trimming of N-linked glycosylation modifications, due to defective P2X_7_ processing in the ER [23].

**Figure 8:**
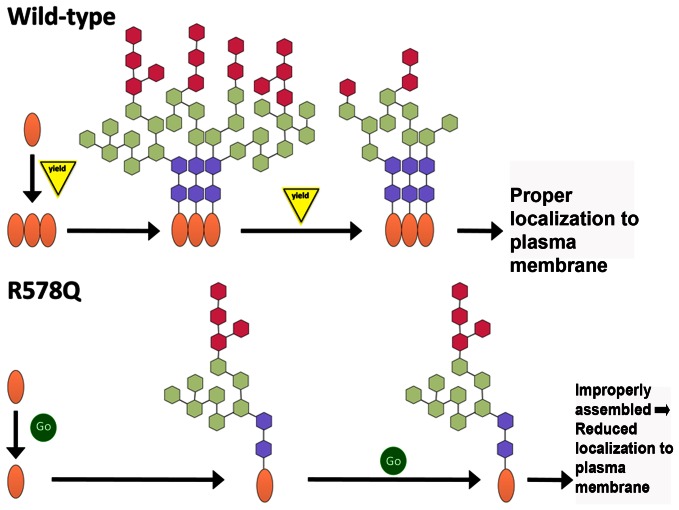
Model of wild-type and mutant P2X_7_ assembly. We propose that P2X_7_ is synthesized in the ER where it is oligomerized and its N-linked glycosylation modifications are added. Mutation of the trafficking domain results in altered kinetics through the secretory pathway, leading to faulty trimerization and insufficient trimming of N-linked glycosylation modifications. Improperly assembled P2X_7_ either does not traffic to the plasma membrane or is quickly removed after it is localized at the plasma membrane. In the model, the protein monomers are indicated by an oval, and the sugar groups are represented by the colored hexagons.

P2X_7_ contains the sequence encoding an Arg-based ER retention signal beginning at amino acid W575 that conforms to the sequence Φ/Ψ/R-R-X-R where Φ/Ψ represents an aromatic or bulky hydrophobic residue and X is any amino acid (Fig. 1a and [18]). Our data demonstrating that mutation of R578 results in reduced activity and impaired oligomerization and modification of N-linked glycosylation is supportive of the idea that P2X_7_ R578Q has an ER protein processing defect. One potential explanation for why mutation of R578 attenuates P2X_7_ activity is that disruption of the R-X-R sequence restricts ER retention, causing P2X_7_ to traffic through the ER before it is properly oligomerized and its N-linked glycans are processed by N-glycan-modifying enzymes. We present several data that indicate P2X_7_ oligomerizes in the secretory pathway and is trafficked to the plasma membrane as a trimer: 1) P2X_7_ trimers are detected on the plasma membrane even in the absence of ligand stimulation (Fig. 3b) and 2) cell-permeable DSS cross-links P2X_7_ monomers (Figs. 4a and 6). There are numerous reports of plasma membrane-bound receptors oligomerizing in the ER [24], [25]. Thus, we propose that both the oligomerization and N-linked glycosylation defects observed for P2X_7_ are attributed to inefficient processing in the ER (Fig. 8). As shown in Figure 6, oligomerization of P2X_7_ appears to be independent of N-linked glycosylation, thus the defect in N-linked glycosylation addition/trimming likely does not contribute to the defect in the ability of P2X_7_ R578Q to trimerize. We postulate that the rate by which P2X_7_ R578Q travels through the ER is altered, potentially through a retention defect, contributing to both the oligomerization and glycosylation problems (Fig. 8).

In contrast to the idea that P2X_7_ contains an R-X-R ER retention signal, mutation of R576 within the Φ/Ψ/R-R-X-R sequence did not result in reduced activity while mutation of R574 attenuated P2X_7_ signaling and pore formation (Fig. 1). These data indicate that if P2X_7_ contains an Arg-based ER retention signal, it does not conform to the Φ/Ψ/R-R-X-R sequence. An alternative explanation to ER retention is that R574 and R578 belong to an ER exit motif. Mutation of R578 to glutamine may prevent P2X_7_ from entering the golgi, allowing for the excessive addition of N-glycans that cannot be trimmed by N-glycan modifying enzymes found later in the secretory pathway. Based on the data presented, we cannot rule out the possibility that oligomerization occurs in the golgi and thus R578 may belong to an ER exit motif.

Because a large portion of P2X_7_ wild-type and R578Q are localized in the ER, as evident by their sensitivity to Endo H (Fig. 5), it is difficult to identify the stage in the secretory pathway where the R578Q defect is occurring. More studies are required to determine whether P2X_7_ R578Q exhibits an ER retention and/or ER exit defect and if its trafficking kinetics through the secretory pathway are indeed altered. A detailed analysis will be needed to identify the specific stage in the pathway where the R578Q variant exhibits its defect. Nonetheless, this investigation demonstrates that the distal C-terminus is critical for normal P2X_7_ activity and that mutation of this region causes decreased receptor function through defective N-linked glycosylation processing, oligomerization, and trafficking to and/or from the plasma membrane.

Our tunicamycin studies revealed the interesting finding that the basal state levels of P2X_7_ monomers are regulated by an N-linked glycosylation-dependent mechanism. As observed in Figure 6, treatment with tunicamycin reduces P2X_7_ protein levels although the cell viability values are similar for cells treated with vehicle, and tunicamycin does not appear to reduce the levels of the loading control vinculin. Thus, it is possible that P2X_7_ synthesis and/or stability are regulated by a protein(s) that is N-linked glycosylated. It would be interesting to characterize the signaling networks that regulate P2X_7_ transcription and protein turnover as this has been largely unexplored. A recent report has shown that the Specificity protein 1 (Sp1) transcription factor regulates P2X_7_ levels in primary cortical neurons and astrocytes [26].

In summary, we provide evidence that human P2X_7_ residues R574 and R578 are required for BzATP-stimulated signaling and pore formation, and that the mutation of R578 to glutamine results in impaired oligomerization and the improper addition and/or trimming of N-linked glycosylation modifications, indicative of a potential processing defect in the ER. To our knowledge, these data are the first to help define the mechanism by which the P2X_7_ distal C-terminus contributes to normal receptor function.
